# The Emerging Field of Nutritional Dentistry

**DOI:** 10.3390/nu14102076

**Published:** 2022-05-16

**Authors:** Johan Peter Woelber, Kirstin Vach

**Affiliations:** 1 Department of Operative Dentistry and Periodontology, Faculty of Medicine, University of Freiburg, Hugstetter Str. 55, 79106 Freiburg, Germany; 2 Faculty of Medicine, Institute of Medical Biometry and Statistics, University of Freiburg, Zinkmattenstr. 6A, 79108 Freiburg, Germany

Nutrition is, like oxygen, one of the basic requirements for animals and, accordingly, Homo sapiens to live. The entire evolution of Homo sapiens was consistently influenced by the nutritional environment, which also included the microbial coevolution. While the most common oral diseases in the form of caries and periodontal diseases were very rare in prehistoric hominins and are still very rare in wild-living animals [1,2,3], today, caries and periodontitis taken together are the most common diseases of mankind [4,5]. Reasons for this dramatic increase can be found in the successive appearance of disease-promoting risk factors in the course of cultural evolution from the Neolithic era to the Industrial Revolution, especially regarding the dietary patterns [6]. This change from a whole-food natural diet towards a Western diet, rich in macronutrients and salt, but poor in fibers and micronutrients, has not only increased the prevalence of oral diseases, but also caused a dramatic increase in non-communicable diseases [7]. Today, diet has even become the strongest risk factor for premature death [8]. On the other hand, these widespread and harmful conditions of a Western diet explains why dietary interventions for oral diseases are so efficient [7,9,10,11,12].

Accordingly, a cause-related prevention and therapy in dentistry must consider these dietary changes in human evolution, which is a fundamental call for the field of nutritional dentistry. This field does not exclude modern symptomatic approaches like plaque control and the use of fluorides [13,14]. It rather aims to build a preventive basis in order to address both oral and other non-communicable diseases in one approach. This strategy is in line with the so-called common risk factor approach [15], which aims to address up-stream risk factors to heighten the efficacy of prevention on an individual and public health level.

Research on this topic demands adequate methods for collecting or generating relevant data as well as for analyzing these data. More and more apps are now being used in addition to the previously widespread paper-based food diaries [16]. In order to achieve comparability of the results, of course, validated survey instruments in several languages are necessary to capture information at different stages across countries and cultures [17]. The statistical analysis of the data also has to meet requirements that go beyond purely descriptive analyses, in order to be able to take the overall complexity into account. In nutritional dentistry, different study types are used, whereas randomized trials of dietary interventions have several limitations and are often unfeasible for ethical or practical reasons, at least as long-term studies. Similar to nutritional epidemiology, there is a broad field of outcomes and exposures.

Against this background, we are honored to add important pieces of evidence for nutritional dentistry by presenting this Special Issue on “Nutrition and Human Oral Health”. It includes twelve studies, two systematic reviews and two narrative reviews. Please let us introduce the articles with a short summary:

Renggli et al. [18] further analyzed data of the Cambodian Health and Nutrition Monitoring Study (CAHENMS) on sociodemographic characteristics, feeding practices and clinical measures for the anthropometric measures and dental status of related Cambodian toddlers. Based on the results, they concluded that severe caries experience was associated with poorer childhood growth and, as such, could be an underinvestigated contributor to stunting.

Components of foods often have special properties, like anti-inflammatory or anti-bacterial effects. Chrubasik-Hausmann et al. [19] investigated the antimicrobial effects of antimicrobial photodynamic therapy (aPDT) with pure juices against typical cariogenic oral Streptococcus pathogens in their planktonic form and its eradication potential on total human salivary bacteria. This pilot study has shown that pure pomegranate juice is superior to the berry juices as a multicomponent PS for killing pathogenic oral bacteria with aPDT. 

In nutritional dentistry research, a basic challenge is to understand the influence of nutrition on the oral microbiota and on the interaction between the oral bacteria, which is also statistically challenging. Thus, Vach et al. [20] investigated log-transformed ratios of two bacteria concentrations as the basic analytic tool. The framework was illustrated by application in an experimental study exposing eleven participants to different nutrition schemes in five consecutive phases. The methods presented made it possible to become independent of the behaviour of other bacteria, which is an advantage compared to common analysis methods of compositions.

Dental patients are often interested in the topic of oil pulling as an additional tool in oral hygiene. In this Special Issue, Kensche et al. [21] investigated the effects of linseed oil on the composition and ultrastructure of the in situ pellicle. They found that linolenic acid was an excellent marker for the investigation of fatty acid accumulation in the pellicle. New preventive strategies could benefit from the accumulation of lipid components in the pellicle.

Dodington et al. [22] determined whether a relationship between periodontal healing and protein intake existed in patients undergoing non-surgical treatment for periodontitis. Dietary protein intake was assessed using the 2005 Block food frequency questionnaire in patients with chronic generalized periodontitis undergoing scaling and root planing. The researchers concluded consuming ≥1 g protein/kg body weight/day was associated with reductions in periodontal disease burdens, following scaling and root planing in patients who were nonsmokers.

In order to identify natural diet-based mouthwashes, Kurz et al. [23] examined the antimicrobial effect of Inula viscosa extract on the initial microbial adhesion in the oral cavity in situ. For the first time, significant antimicrobial effects on the initial microbial adhesion in in situ oral biofilms were reported for an I. viscosa extract.

Since healthy diets and their associations might not only rely on one dietary factor, it is important to investigate their association with regard to complete dietary patterns. Within this context, Altun et al. [24] investigated the relationship between specific known dietary patterns and the prevalence of periodontal disease in a northern population-based cohort study. They evaluated data from 6209 participants of the Hamburg City Health Study (HCHS). The current cross-sectional study identified a significant association between higher adherence to the DASH and Mediterranean diets and lower odds of being affected by periodontal diseases (irrespective of disease severity).

Ketogenic diets are dietary patterns, which almost completely exclude carbohydrates. They’re used for a variety of diseases and corresponding dietary therapies like epilepsy, forms of cancer, obesity, or diabetes. Woelber et al. [25] investigated the safety and the effect of a ketogenic diet on oral parameters. They found that the ketogenic diet did not lead to clinical changes in periodontal parameters in healthy participants under continued oral hygiene, but it did lead to a significant weight loss.

Since the relationship between low-density lipoprotein cholesterol (LDL-C) or levels of 25-hydroxyvitamin D (25OHD) with periodontal diseases is frequently discussed in the literature, Thim et al. [26] investigated the association between these serum parameters and radiographic levels of bone loss in 163 dental patients. They found that radiographic bone loss (RBL) was associated with known patient-specific markers, particularly with age and high LDL-C levels. Patients with high 25OHD levels (≥40 ng/mL) exhibited significantly less RBL.

A further important topic in this area is the assessment of dietary behavior in relation to oral health. Schlenz et al. [27] investigated whether traditional dietary questionnaires are suitable for assessing the relationship between tooth wear and diet. They found that none of the assessed dietary parameters showed a significant relationship with tooth wear and concluded that the suitability of dietary questionnaires to assess tooth-relevant dietary behavior seems to be limited. Bartha et al. [28] analyzed data of a randomized clinical trial to assess the usefulness of the Mediterranean Diet Adherence Screener (MEDAS) with regard to periodontal parameters. They found that MEDAS was a sufficient diet correlating with oral inflammatory parameters. Due to this, the MEDAS might also be useful in dental practice.

Nicklisch et al. [29] performed a bioarchaeological study on caries and stable isotope data obtained from prehistoric individuals (*n* = 101) 17 from three Early Neolithic sites (c. 5500-4800 BCE) in central Germany. The combined evidence from caries and isotope analysis suggested a prevalence of starchy foods, such as cereals, in the diet of these early farmers.

Even though these studies represent a rather small sample, one can already gain an impression of the efficacy and power of nutritional dentistry. Furthermore, this field is still quite uninvestigated, with a lot of unknown helpful knowledge which could empower everyone’s lives in regard to oral and overall health. This also applies to the pathogenicity of oral biofilms (dental plaque) and the corresponding host resistance, whereby initial nutritional studies no longer see any or only a very weak correlation between plaque and oral diseases, like caries and periodontal inflammation, in natural or optimized dietary environments [9,10,11]. Future studies will also have to consider the application and development of new statistical methods in order to deliver a sufficient picture of the connections [20,30].

Based on the impressive studies of this Special Issue, we hope to have made a small contribution to the emerging field of nutritional dentistry.
